# The immune-modulatory role of MSCs exerted by PI3K/AKT signaling pathway in kidney tissue after cyclophosphamide

**DOI:** 10.4314/ahs.v23i4.40

**Published:** 2023-12

**Authors:** Heba M Saad Eldien, Mohammed Jayed Alenzi

**Affiliations:** 1 Department of Anatomy, College of Medicine Al-Jouf University; 2 Al Jouf University, Surgery

**Keywords:** Cyclophosphamide (CP), kidney, PI3K, AKT, CD21, CD14, bone marrow-derived MSCs

## Abstract

**Background:**

Cyclophosphamide (CP) is one of the most effective immunosuppressive agents. To understand the mechanisms used by the CP and MSCs in the kidney, we investigated their effects on some pathways.

**Experimental animals and methods:**

4 groups of female rats were used. GI: was the normal control group treated with saline solution. Groups G II, G III, and G IV were treated with CP. G I and G II groups were sacrificed on the fourth day after treatment., G III (Auto healing group) was left without treatment after the CP injection for six days. The G IV group was treated with MSCs on the fourth day after the CP injection. G III and G IV groups were sacrificed six days after treatment, and the kidney was removed and processed.

**Results:**

CP induced up-regulation in CD14 and CD21 positive cells and caspase. Significant down-regulation of previous markers in groups III and IV. CP exerted a downregulation effect on AKT/ PI3K, that were ameliorated in groups III and IV. A significant increase in P53, BCL2, as well as VEGF in Group IV (P < 0 05).

**Conclusion:**

MSCs play a vital function in the immune inhibition in CP-treated rats through PI3K/AKT pathway.

## Introduction

Cyclophosphamide (CP) is an alkylating substance utilized as an anticancer and immunosuppressive drug 1. Minimizing immunosuppressant-induced nephrotoxicity is a great challenge. Other challenges include post-transplant rejection, especially in the long run, and finding out immunosuppressive therapies with relatively few side effects. Immunosuppressives might help in improving long-term renal allograft survival. However, to improve significantly renal allograft outcomes, it may be necessary to adopt new immunosuppressive methods 2. MSCs have the characteristics of self-renewal and trilineage differentiation potential; they can differentiate into the mesoderm, ectodermal and endodermal lineages 3. MSCs can repair the ischemic renal tubular injury in conditions of renal ischemia-reperfusion injury through several mechanisms, such as anti-inflammatory, angiogenic, anti-apoptotic, and anti-fibrotic effects, as well as secretory cytokines and their paracrine and endocrine effects.

Moreover, immunoregulatory might help in renal tissue regeneration. In vitro and in vivo findings revealed the immunosuppressive properties of MSCs 4. The immunomodulatory properties of MSCs are either stimulating or down-regulating the immune response. The proven immunomodulatory mechanism of action of MSCs might explain their effectiveness. Some clinical trials confirmed these effects in cases of graf-versus-host disease (GVHD) 2. Thus, we can say that the MSCs' application might potentially impact and solve the problem of the inevitable use of immunosuppressive drugs in kidney transplantation, or at least reduce their doses, with the enhancement of the renal function of patients, reducing opportunistic infections, and improving their long-term survival.

Reducing nephrotoxicity in recipients of transplanted organs is essential to reduce substantial savings in health care costs and patient morbidity and mortality 5. Furthermore, combined treatment with bone marrow-derived MSCs and immune suppressants might minimize acute renal rejection and protect against renal tubular atrophy for 24 weeks after MSC treatment. Thus, MSCs injection might exert a potential stabilizing effect on renal graft function. Moreover, MSC application might help in a significant reduction of immunosuppressive doses. These effects might be due to inhibiting T-cell mitosis and minimizing graft inflammation. Moreover, multiple sources of stem cells might exert a synergistic effect in providing more protection for renal grafts 5.

## Materials and methods

### Investigational animals

Female Wistar rats five to six months old, weighing 180-200 g, were acquired from the Laboratory Animal House of the Faculty of Medicine, Assiut University, Egypt.

Animals were kept for one week prior to the experiment for acclimatization, then housed in polycarbonate cages (six rats/cage) (cages with three levels, each level is four cubic feet to ensure two cubic feet of space per rat). The animals were maintained in a controlled laboratory environment at a constant temperature (22-24°C) and a light-controlled room on an alternating 12:12 h light-dark cycle. Access to drinking tap water was freely allowed for rats. During the experiment, a standard pellet diet was given. The animals matched the Institute of Laboratory Animal Research regulations for experimental animal work 6. Ethical approval was granted after revision from the Medical Ethics Committee in the Faculty of Medicine, Assiut University (IRB 17300339).

### Experimental model and treatment protocols

Twenty-seven rats (three males and twenty-four females) were used in this study. The three male rats were served for isolation of MSCs. The twenty-four female rats were equally divided into four groups. Rats in the normal control group (G I) were diurnal and administered sterile saline intraperitoneally for four days. The other three groups of rats (G II, G III, and G IV) were treated with intra-peritoneal cyclophosphamide at 70 mg/kg BW/d for three days. Then, a single dose of male BM-derived MSCs 5X106/kg BW was given to the G IV group on the fourth day by intraperitoneal injection. The G III (auto-healing group) was left without treatment after the CP injection. Animals in G I and G II groups were sacrificed on the fourth day after CP treatment. Six days later, groups G III and G IV animals were sacrificed. Scarification was performed after induction of general anesthesia with propofol (4 mg/kg/i.v., Propovet®, Abbott Laboratories, Kent, UK). Dissection of animals and sampling were performed in an animal operating theatre under aseptic conditions. The kidney was removed and processed for light microscopy examination, and samples were stored at -80°C for RNA extraction.

### Histological analysis

Kidneys were extracted and placed in 10% neutral buffered formalin for 24 h. A Routine paraffin procedure was used. After paraffin embedding of renal tissues, sectioning and staining were performed then examination was performed by an upright light microscope 7.

### Isolation and culture of rat BM-MSCs

Eight weeks old male rats were sacrificed and kept in a 100-mm cell culture dish (Becton Dickinson, Franklin Lakes, NJ, USA). Then their bodied were immersed in 70% (v/v) EtOH for 2 min. Then the mouse is transferred to another sterile plate. Removal of skin and dissection was performed. All soft tissues were removed to avoid contamination. Then bone was transferred to a 100-mm sterile culture dish with 10 mL α-MEM medium(Gibco™ A1049001). Rapid processing was conducted within 20 min after animal death to ensure the maximum viability of cells. In a biosafety cabinet, the bones were washed with PBS having 5% penicillin/streptomycin/amphotericin B (5,000 U/m, ThermoFisher - Catalog number: 15070063). Then the skeletons were kept in a fresh 100-mm sterile culture dish with 10 mL whole a-DMEM medium. A 3 mL syringe was used for flushing marrow cavities several times until the bones became pale. The dish was incubated at 37°C in a 5% CO_2_ incubator. The first recognizable spindle-shaped cells seemed on the third day using inverted microscopy; within 7-8 days, MSCs achieved 70–90% confluence. Cells are double-washed with PBS and trypsinized. Then phenotypic characterization of the rat BM-MSCs at passage 2 was conducted. MSCs characterization was performed using flow cytometry, mesenchymal stem indicators (CD90 and CD73), and hematopoietic indicator CD45 8.

### Immunohistochemical assay

The slides were placed at 56-60°C in an oven for 20 min and then de-paraffinized and washed by rinsing the slides in gently running tap water for 40 seconds and then the slides were kept in a PBS bath for further rehydration (30 min at room temperature). Antigen retrieval was conducted by using a microwave and citrate buffer. Then tissue sections were incubated in 3% H2O2 (K31355100, Merck, Darmstadt, Germany) for 4 min. After that, tissue sections were incubated with appropriate primary antibodies (anti-CD14) (1:500) (Abcam), anti-follicle dendritic cell markers (anti-CD21) (1:400) (Abcam), anti-protein kinase B (anti-AKT) (1:400) (Abcam), and anti-phosphoinositide 3-kinases (anti-PI3K) (1:400) (Abcam) antibodies using the producer's guidelines, applying the Horseradish peroxidase/DAB (ABC) recognition immunohistochemistry kit (Abcam, UK). A brownish color identifies the antigen-positive area. The tissue sections were incubated with secondary antibody (85-9043; Invitrogen, Camarillo, CA, USA) overnight at 4°C and then with strepavidin (85-9043; Invitrogen, Camarillo, CA, USA) in PBS for 25 min each. In addition, the tissue slides were washed with PBS three times and incubated with diaminobenzidine (ACK125, ScyTek DAB Chromogen, Logan, Utah, USA) for 10 min before counterstaining with Mayer's hematoxylin (MHS16 Sigma-Aldrich, USA). Then tissue sections were dried, cleared, and mounted with glycerol gelatin, and coverslips were used. Quantitative analyses of CD14, CD21, AKT, and PI3K protein expressions after immunohistochemical staining by the previous four markers using ImageJ, the percent coverage of immunostaining of four markers were quantified to eventually compare differences in four groups. We removed the counterstained nuclei from the image using the Threshold Colour plugin in order to only assess the positive DAB staining. 9

### Real-time quantitative PCR (RT-qPCR)

RNA extraction was performed by applying TRIZOL (Invitrogen, CA, USA) using the producer's instructions (12). cDNA creation was conducted using Extra-Capacity cDNA Reverse Transcription Kit (ThermoFisher Scientific, Inc) according to the producer's guidelines. The expression of P53, BCL-2, caspase-3, vascular endothelial growth factor (VEGF), and SRY genes was assessed by RT-qPCR. GAPDH was used as a housekeeping gene. Primer sequences are presented in Table 1. The RT-qPCR was conducted in a StepOne real-time PCR machine (Functional Biosystems, Foster City, CA) (10) as observed: 1) 94°C for 2 min and 2) magnification over 40 cycles at 94°C for 15 s and at 58–62°C (based on the primer put) for 30 s. Data were evaluated via Sequence Revealing Software version 1.3.1 (Applied Biosystems).

**Table 1:** Sequences of the primers

Primer	The primer's Sequence
***P53***	Forward: 5′- GTTCCGGAGCTGAATGAGG -3′ Reverse: 5′ – TTTTATGGCGGGACGTAGAC -3′
***BCL2***	Forward: 5′-TGAACCGGCATCTGCACAC-3 Reverse: 5′-CGTCTTCAGAGACAGCCAGGAG-3′
***Caspase3***	Forward: 5′- CGGAGCTTGGAACGCGAAGA-3′ Reverse: 5′- ACACAAGCCCATTTCAGGGTAA-3′
***VEGF***	Forward: 5′- GTGCACTGGACCCTGGCTTT-3′ Reverse: 5′- CCCTTCTGTCGTGGGTGCAG-3′
***SRY***	Forward: 5′-AGGGTTAAAGTGCCACAGAGGA-3′ Reverse: 5′-GCTTTT CTGGTTCTTGGAGGAC-3′.
***GAPDH***	Forward: 5′-AACCTGCCAAGTATGATGACATCA-3′ Reverse: 5′-TTCCACTGATATCCCAGCTGCT-3′

## Results

### Histological findings

**Figure 1:**
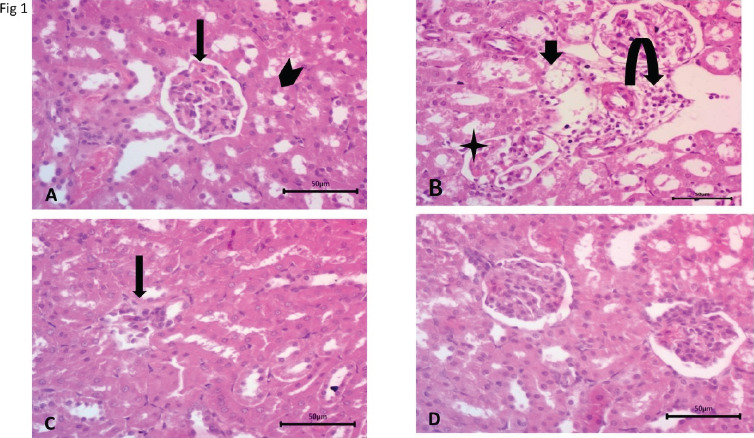
A) Photomicrograph of a cross-section of the cortical locality of the kidney of the control represents renal corpuscles (black arrow) and convoluted tubules (arrowhead) B) After cyclophosphamide treatment showing degenerative changes in kidney tubules (black arrow) and wide Bowman's space (star). Mixed inflammatory cells infiltrated close to the renal corpuscle and between kidney tubules (curved arrow) C) In the auto-healing group, some inflammatory cells infiltrated between tubules D) After MSCs treatment, no pathological changes occurred. (H&E stain) scale bar, 50 µm

The photomicrograph of normal renal tissue presented the cortical zone with the renal corpuscles shaped by the round frame of glomeruli alienated from encompassing assemblies by Bowman's space. Multiple sections of convoluted tubules were satisfactorily outlined and detached by ordinary interstitium (Figure 1A).

After cyclophosphamide treatment, degenerative changes in kidney tubules were observed along with a wide Bowman's space, and mixed inflammatory cells infiltrated close to the renal corpuscle and between the kidney tubules (Figure 1B).

In the auto-healing group, some inflammatory cells' infiltrate was observed between tubules with no observed pathological features (Figure 1C). After MSCs' treatment, no pathological changes were perceived at the renal particles and the interstitium (Figure 1D).

### Immunohistochemical assay

**Figure 2:**
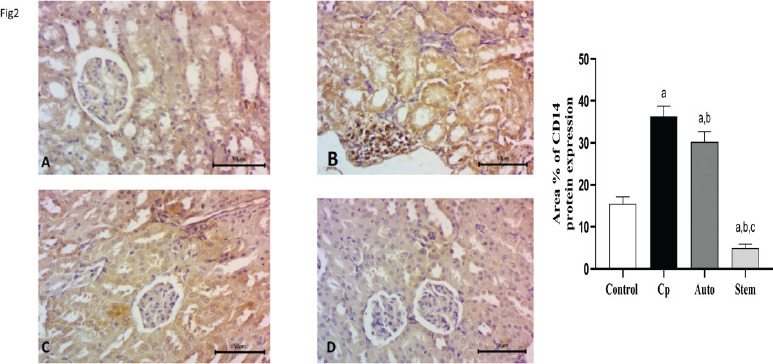
**A, B, C, d, E).** Expression of CD14 in rat renal tissue. Herein, we evaluated the quantity of CD14 positive cells in the glomeruli and renal tubules by immunohistochemistry. It was significantly up regulated in the cyclophosphamide treated group, and significantly downregulated in auto healing group and high significant down-regulation after MSCs treatment in comparison to CP treated group, Magnification, x400. scale Bar 50um

Expression of CD14 in rat renal tissue as a cell indicator of macrophages. Herein, we evaluated the quantity of CD14-positive cells in the glomeruli and renal tubules by immunohistochemistry. CD14 expression in the glomeruli and renal tubules was up-regulated in the cyclophosphamide-treated group and significant down-regulated in auto healing group and high significant down-regulation after MSCs treatment in comparison to CP treated group, (Fig. 2 A, B, C, d, E).

**Figure 3:**
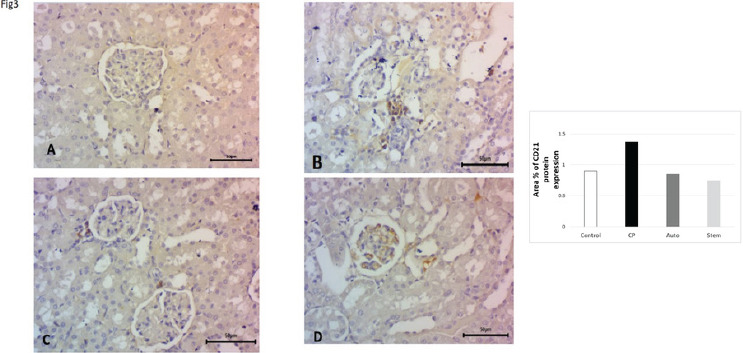
**A, B, C, d, E:** Expression in rat renal tissue CD21. Herein, we evaluated the quantity of CD21-positive cells in the glomeruli and renal tubules by immunohistochemistry. It was up regulated in the cyclophosphamide treated group, and significantly downregulated in auto healing group and high significant down-regulation after MSCs treatment in comparison to CP treated group. Magnification, x400.scale Bar 50um

Expression of CD21 in rat renal tissue as a cell surface indicator of the follicular dendritic cell. Herein, we studied the quantity of CD21-positive cells in the glomeruli and renal tubules by immunohistochemistry. CD21 expression in the glomeruli and renal tubules was up-regulated in the cyclophosphamide-treated group, and significant down-regulated in auto healing group and high significant down-regulation after MSCs treatment in comparison to CP treated group (Fig. 3 A, B, C, d, E).

**Figure 4:**
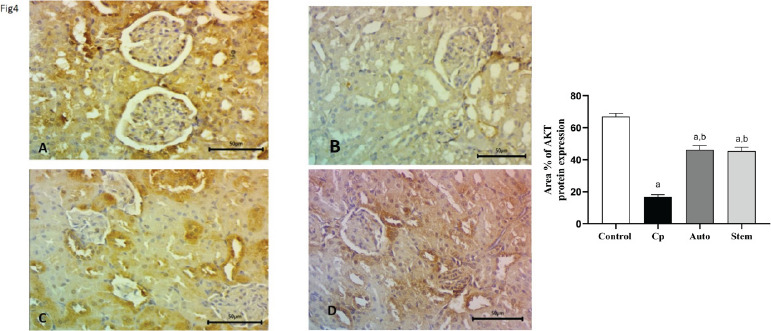
**A, B, C, d, E):** Expression in rat renal tissue AKT kinase, Herein, we evaluated the quantity of AKT-positive cells in the glomeruli and renal tubules by immunohistochemistry. It was down-regulated in the cyclophosphamide-treated group, and significantly up-regulated in auto healing group and after MSCs treatment in comparison to CP treated group. Magnification, x400.scale Bar 50um

Expression of AKT protein in rat renal tissue. AKT/PI3K kinas controls several procedures namely metabolism, proliferation, cell existence, growth, and angiogenesis. In our study, we evaluated the quantity of AKT-positive cells in the glomeruli and renal tubules by immunohistochemistry. AKT expression in the glomeruli and renal tubules was down-regulated in the cyclophosphamide-treated group, and significantly up-regulated in auto healing group and after MSCs treatment in comparison to CP treated group (Fig. 4 A, B, C, d, E).

**Figure 5:**
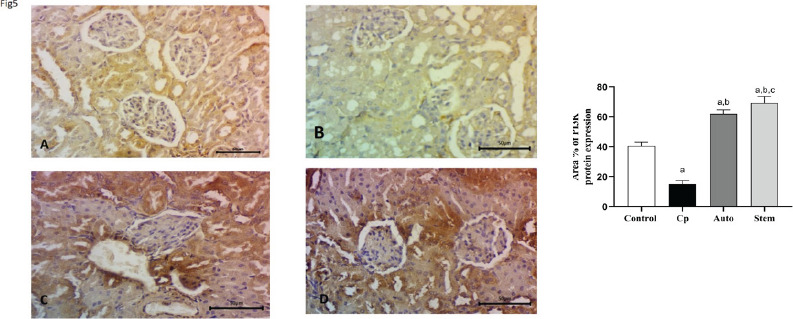
**A, B, C, d:** Expression in rat renal tissue PI3K. Herein, we evaluated the quantity of PI3K -positive cells in the glomeruli and renal tubules by immunohistochemistry. It was downregulated in the cyclophosphamide-treated group, with significant upregulation in the auto-healing group and after MSCs treatment in comparison to CP treated group Magnification, x400.scale Bar 50um

Expression of PI3K protein in rat renal tissue. We evaluated the quantity of PI3K-positive cells in the glomeruli and renal tubules by immunohistochemistry. PI3K expression in the glomeruli and renal tubules was significant downregulated in the cyclophosphamide treated group, and significant upregulated in the auto-healing group and after MSCs treatment in comparison to CP treated group (Fig. 5 A, B, C, d, E).

Immunohistochemistry (IHC) analyses of CD14, CD21, AKT, and PI3K protein expressions in kidney tissues of the 4 groups of rats. Quantitative evaluations of CD14, CD21, AKT, and PI3K illustration as determined by IHC. The number of CD14 + macrophages and CD21 positive dendritic cells were significantly upregulated and downregulated after treatment with cyclophosphamide and MSCs respectively. AKT, PI3K positive cells were significantly down-regulated and up-regulated after treatment with Cyclophosphamide and MSCs respectively

### qPCR outcomes

**Figure 6:**
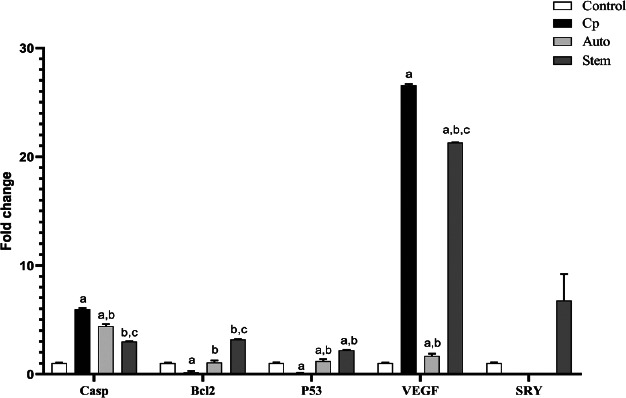
mRNA expression of caspase, BCL2, P53 and VEGF in the kidney tissue of the control, cyclophosphamide treated, auto healing and MSCs treated groups, mRNA expression of SRY gene indicating of Y chromosome-linked gene confirms the presence of male-derived MSCs in kidney tissue derived from female rats (P < 0.05)

Significant up-regulation of the mRNA expression of caspase and VEGF in the kidney tissue of the cyclophosphamide-treated group was associated with a significant reduction in BCL2 and P53 mRNA expression, in comparison to control animals (p<0.001). After MSCs treatment, significant down-regulation of caspase was associated with significant up-regulation in mRNA of BCL2, P53 (p<0.001). Whereas non-significant up-regulation of VEGF, in comparison to CP treated group(p>0.05). In the auto-healing group, a significant reduction of caspase expression in comparison to the CP-treated group. (p<0.05), as well as significant increase of BCL2 and P53 expression in comparison to the CP-treated group(p<0.001). Also, a significant decrease was observed in the mRNA expression of VEGF in the auto-healing group, in comparison to CP treated group (p<0.05). The observed significant up-regulation of mRNA expression of SRY gene (Y chromosome-linked gene) in MSCs treated group, confirms the presence of male-derived MSCs in kidney tissue derived from female rats (P < 0.05) (Figure 6). In cyclophosphamide-treated animals enhance the macrophage phagocytic as well as dendritic cell activity. (Figures 2&3) Overall, these outcomes propose that MSCs play a vital function in the shield versus immune inhibition in cyclophosphamide-treated rats and might be a possible nominee for immune treatment regimens.

**Figure 7:**
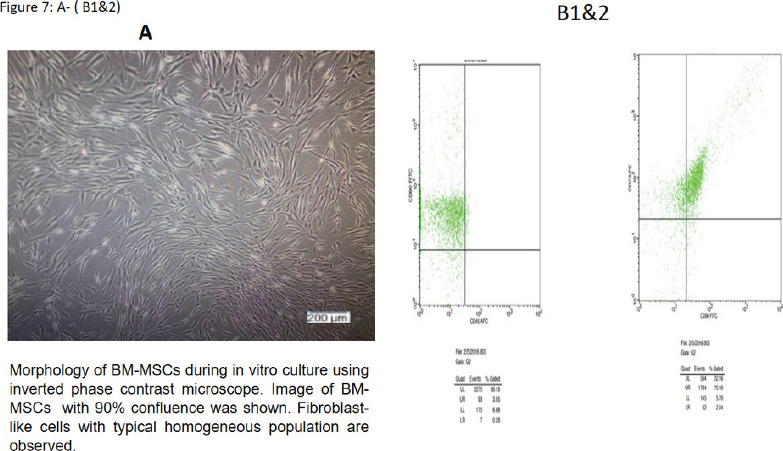
**A:** Morphology of BM-MSCs during in vitro culture using inverted phase contrast microscope. Image of BM-MSCs with 90% confluence was shown. Fibroblast-like cells with typical homogeneous population are observed. B1&2. Phenotypic characterization of the rat BM-MSCs using flow cytometry. Mesenchymal stem markers (CD90, CD73) were positively expressed and hematopoietic marker CD45 was negatively expressed.

Morphology of BM-MSCs during in vitro culture using an inverted phase contrast microscope revealed fibroblast-like cells with a typical homogeneous population, cells appeared in 90% confluence. (Figure 7 A)

Phenotypic characterization of the rat BM-MSCs using flow cytometry. Mesenchymal stem markers (CD90, CD73) were specified, and depression in hematopoietic indicator CD45 (Figure 7 B1&2)

## Discussion

Cyclophosphamide (CP) is one of the most effective anticancer agents. It is a potent immunosuppressive drug used to treat autoimmune disorders 11. It enhances the antitumor action of immunotherapy. Increasing demands for understanding the base of cyclophosphamide-mediated immunomodulation are crucial to expanding the efficiency of chemoimmunotherapy. Positive bindings between chemo- and immunotherapy is vital to enhance synergisms between both types of treatment. Previous studies reported the ability of CP to enhance immunity, possibly through its activation of the p53 signaling, its cytotoxicity and resulting DNA impairment, and cell-cycle arrest 12.

The increase in apoptotic changes upon the cytotoxic effect of CP might explain the observed increase in CD14 and CD21 positive cells after CP treatment. These effects might stimulate and enhance the chemotaxis of phagocytic cells in the degenerated renal tissue. These suggestions are raised and confirmed by the qPCR data, as there is a significant up-regulation of mRNA expression of caspase in the renal tissue in the cyclophosphamide-treated group. Several authors support our suggestions as they observed increased expression of antigen-administering mediators, up-regulation, and split initiation of a monocyte subset CD14 following cyclophosphamide treatment.

Moreover, CD16-expressing cells are considered by macrophage-similar characteristics, strong endocytosis effects, and high antigen presentation function associated with the excess making of pro-inflammatory cytokines 13. Previous studies explained amplified fractions of these cells by induction of phagocytic/activated monocytes that might help in phagocytosis of neighbouring dead cells after cyclophosphamide treatment associated with a lack of these cells' responsiveness to cyclophosphamide cytotoxicity 14. Following these findings, others confirmed the immune-modulatory role of cyclophosphamide by induction of the extension/beginning of CD14 and CD16 monocytes, as well as HLA-DR, IL-8RA, and MARCO monocytes/dendritic cells 12.

The observed reduced CD14 and CD21 expression after MSCs treatment might be explained by their anti-inflammatory effect that reduces monocyte and dendritic cell expression and activation. Others supported these suggestions and explained the MSCs' immunosuppressive effects by their apoptosis-enhancing effects on inflammatory cells. Considerable evidence was previously reported in vitro that MSCs could conquer the development of monocyte-resulting dendritic cells. Dendritic cells (DCs) are the main potent antigen-presenting cells that perform crucial functions in orchestrating cellular and humoral immunity. DCs might be crucial in differentiating naïve T-cells into T-helper-1 or T-helper-2 effecter cells 15.

To understand the mechanisms used by the cyclophosphamide and MSCs in the kidney, we investigated the effects on PI3K/AKT signaling pathways. The expression of PI3K and AKT in rats treated with cyclophosphamide decreased significantly, equated with the control (p < 0.01). These results indicated that CP inhibits PI3K/AKT signaling pathways. Meanwhile, it was significantly up-regulated by the MSCs' treatment but non-significantly up-regulated in the auto-healing group.

In our study, a significant decrease in PI3K and AKT expression in cyclophosphamide–treated animals suggests that cyclophosphamide's mechanisms of action might be exerted through the downregulation of the PI3K signaling pathway. The PI3K/AKT signaling pathway plays a vital role in cell growth, proliferation, and existence. The downstream marks of the PI3K/AKT/mTOR signaling pathway contain protein kinase mTOR, the most vital controller of translation and autophagy. So far, few studies have confirmed the association of renal diseases with PI3K/AKT upregulation. Some studies suggested that PI3K/AKT may play a vital role in many renal syndromes 16.

Nevertheless, experimental inhibition of PI3K-AKT signaling pathways could significantly minimize extracellular matrix accumulation in damaged kidneys. This emphasizes the significance of the PI3K/AKT pathway as a vital controlling factor of renal fibrogenesis, adjustment of fibroblast initiation, proliferation, and relocation, and ECM deposition after renal damage. It was found that several anticancer drugs have a potential interference with PI3K/AKT pathways. Moreover, some of these drugs can be utilized to avert renal fibrosis and as a treatment for renal-failure. Recent studies further supported the participation of the PI3K-AKT signaling pathway in renal mutilation 17. According to these studies, the down-alteration of the PI3K-AKT signaling pathway might be accountable for reducing pro-fibrotic interstitial cells and excessive interstitial matrix production. Therefore, activating AKT might play a crucial role in normalizing hyperplasia of renal cells, as well as fibroblast function and carrier construction in chronic kidney disease.

However, the importance of AKT activation in activated fibroblasts might explain the pathophysiological procedures of wound medicine. Thus, AKT signaling might be a remedial goal for treating renal damage induced by cytotoxic agents. Moreover, it has been found that up-regulating AKT/PI3K by MSC transplantation or MSC genetically overexpressing AKT exerted cardiomyocytes' regenerative effect by significantly enhancing intramyocardial retention 18. Consequently, initiating AKT requires several cellular procedures, for example, proliferation, existence, growth, metabolism, and angiogenesis. This is facilitated across serine or threonine phosphorylation of downstream components.

It has been found that MSCs induced AKT/PI3K enhancement effect; this effect might be exerted through VEGF up-regulation. These suggestions were raised after the work of 19, who explained the therapeutic effect of MSC in diabetes by its vascular endothelial evolution factor over-expressed and initiation of PI3K/AKT/mTOR/eNOS and p38/MAPK signaling pathways, suggesting a critical role of MSC-derived packed proteins in the renal regenerative. The anti-fibrotic effects of MSCs compensate for possible pro-fibrotic effects that might result from the beginning of the MAPK pathway.

The observed VEGF up-regulation in the present study after CP treatment was explained by previous studies by the up-regulation of cyclophosphamide-induced cyclooxygenase-2 (COX-2) and many inflammatory facilitators in the urinary bladder after CP-generated cystitis in rats 20, 21, 22. Vascular endothelial growth factor (VEGF) is a pleiotropic cytokine with angiogenic and high vascular penetrability-boosting potential 23. Numerous factors might regulate its expression in various tissues, including hypoxia, inflammatory mediators, hormonal milieu, COX-2, and mechanical strengths, and in tumors by oncogenes and tumor suppressor genes 24.

The observed vascular endothelial growth factor (VEGF) secreted by MSCs in renal tissue was examined via reverse transcription-quantitative polymerase chain reaction to perceive the articulation of genes. VEGF re-upregulation after MSCs treatment might be explained by their AKT/PI3K-inducing effects 18.

The observed MSCs' stimulating effect for P53 expression after the exerted CP' down-regulating effect might have a beneficial effect on cell proliferation, relocation, differentiation, and the cell cycle, maintaining the genome integrity. P53 is measured as a tumor suppressor that maintains genome integrity by regulating numerous cellular processes. P53 may also utilize its fundamental purposes by adjusting DNA methylation 25. Its function as a transcription factor that acts as a cell homeostasis regulator 26 might help in numerous cellular processes, such as cell cycle control, growth, differentiation, and DNA repair. Accordingly, P53 is considered the protector of the genome 27. Loss or mutation of P53 expression happens in about 50% of human cancers 28, which might cause genome uncertainty and functional abnormalities in the cell cycle, proliferation, migration, and differentiation.

A similar observation was detected after MSCs' treatment of irradiated mice, whereas amplified P53 articulation was noticed by immunohistochemistry in thymus tissues 29. Therefore, using MSC induced the expression of the anti-apoptotic gene BCL2. However, it down-regulated the articulation of the pro-apoptotic gene's caspase in CP-treated kidney tissue, signifying that alteration of programmed cell fatality may lead to MSC-induced renal repair. A superior estimation of P53-facilitated apoptosis and P53-facilitated tumor containment typically supports the potential for numerous possible clinical claims. Thus, these MSCs-induced effects might improve the proficiency of anticancer treatments that rely on P53 initiation, dropping the toxicities related to chemotherapy or radiotherapy and expanding stem cell transplant acclimatizing treatments.

Moreover, in non-malignant conditions such as myocardial infarction and cerebral ischemia, up-regulation of cell death pathways (P53 driven) precipitate tissue damage 30. Meanwhile, we found that the correlation between MSCs' immunosuppressive effects (down-regulation of CD12 and CD21 cells) and MSCs-induced AKT/PI3K enhancement effect, as well as VEGF upregulation, raise the suggestion that AKT/PI3K signaling pathways might be implicated in the immunosuppressive effects of MSCs through VEGF upregulation.

The results confirmed that MSCs could protect kidney structure and function against CP-induced injury through antioxidant, anti-inflammatory, and anti-apoptotic activities.

## Conclusion

The results of the present study propose that MSCs avert the development of CP-induced renal toxicity by a mechanism linked, at least in part, to its capability to spread mRNA articulation of antioxidant genes and to decline apoptosis in renal tissues with the resultant upgrading in AKT/PI3K signaling pathway. Moreover, MSCs play a vital function in the shield versus immune inhibition in CP-treated rats through PI3K/AKT signaling pathway by Increasing VEGF Production.
